# Spectral homogeneity of human platelets investigated by SERS

**DOI:** 10.1371/journal.pone.0265247

**Published:** 2022-05-11

**Authors:** Andrey Zyubin, Vladimir Rafalskiy, Mikhail Lopatin, Elizaveta Demishkevich, Ekaterina Moiseeva, Karina Matveeva, Igor Kon, Artemii Khankaev, Anna Kundalevich, Viktoria Butova, Leonard Lipnevich, Ivan Lyatun, Ilia Samusev, Valery Bryukhanov

**Affiliations:** 1 REC «Fundamental and Applied Photonics, Nanophotonics», Immanuel Kant Baltic Federal University, Kaliningrad, Kaliningrad Region, Russia; 2 REC «Clinical Trials Center», Immanuel Kant Baltic Federal University, Kaliningrad, Kaliningrad Region, Russia; 3 Institute of Physics, Saratov State University, Saratov, Russia; Ludwig-Maximilians-Universitat Munchen, GERMANY

## Abstract

This paper describes a detailed study of the spectral homogeneity of human platelets using Surface-enhanced Raman spectroscopy (SERS). We used a combined approach based on multivariate methods as principal component analysis and pair correlation algorithms to investigate platelets spectral properties. The correlation coefficients for each sample have been calculated, and the average coefficient of determination has been estimated. The high degree of spectral homogeneity inside one probe and between them has been revealed. The prospects of obtained results usage for pathologies based on platelet conformations during cardiovascular diseases have been demonstrated.

## Introduction

Cardiovascular deceases (CVD) have remained the leading cause of death at the global level for the last two decades worldwide. CVD total cases were doubled from 271 million in 1990 to 523 million in 2019, and the number of CVD deaths increased from 12.1 million in 1990, reaching 18.6 million in 2019 [1]. The process of thrombus formation plays a crucial role in the detection, diagnosis, and treatment of CVD, and it is the object of close interest for scientists of the whole world [2, 3]. The platelet and its structural changes investigations under the influence of internal and external factors still remain a challenging task today [4, 5]. The platelet activation is considered to be a key moment in the pathogenesis of CVD and its complications; therefore, the inhibition of the platelet aggregation-responsible receptors is the most important task in the course of treatment and prevention of CVD. The variability of response to antiplatelet therapy is also an important topic for the study and successful antithrombotic therapy [6]. Raman spectroscopy and SERS can be very informative for platelets molecular structure investigation [7]. Raman-based spectroscopy methods used to perform the analysis of the molecular components such as amino acids, proteins, lipids, etc. [8, 9] can bring the understanding of the platelet structure and its spectral response to the antiplatelet therapy, which is the key to personalized medicine. Despite the fact that all platelets are heterogeneous in size, age, and responsiveness to different activators and morphologically and functionally different platelet subpopulations participate in the spatiotemporal regulation of hemostatic plug and thrombus formation [10, 11] the platelet *spectral* homogeneity (or heterogeneity) is still an important question today. Insofar as every decrease is associated with a change in biochemistry, the uniformity of the platelet spectra in a particular person can be used as a reference point to assess changes in the molecular structure of patients in case of CVD complications, platelet activation, or drug antiplatelet therapy control.

Our paper performs new results for platelets spectral homogeneity analysis using SERS and statistical methods usage to analyze it for the healthy person. As a result, the human platelets spectral homogeneity was demonstrated.

## Materials and methods

### Subject

15 blood samples were taken from one healthy volunteer in 6 different days within two-week period. Written informed consent had been obtained from healthy volunteer before any study procedures. All study documents including informed consent and protocol were approved by Immanuel Kant Baltic Federal University Independence Local Ethic Committee (Protocol № 8, 16.05.2019). Healthy volunteer was 35 years old and had no acute and chronic diseases. The patient did not smoke or take any antiplatelet or anti-inflammatory drugs.

### Platelets extraction

We used preparation protocol was based on [12] and previously described in [8] Briefly, fresh venous blood samples were taken from healthy volunteer in vacuum tube containing EDTA (BD Vacutainer® spray-coated K2 EDTA Tubes). It was centrifuged at 60 g for 15 min to consequentially separate platelet-rich plasma (PRP) from red blood cells (RBC) and leukocytes. After that, the PRP was collected and placed to the new tube. Platelets were finally collected by further centrifugation of the supernatant at 1500 g for 15 min. All the centrifugations were carried out at 4°C using Eppendorf 5702R centrifuge. After platelet preparation the samples were immediately taken to be examined by Surface-enhanced Raman spectroscopy (SERS).

### SERS substrates fabrication and simulation experiments

**Fig 1:**
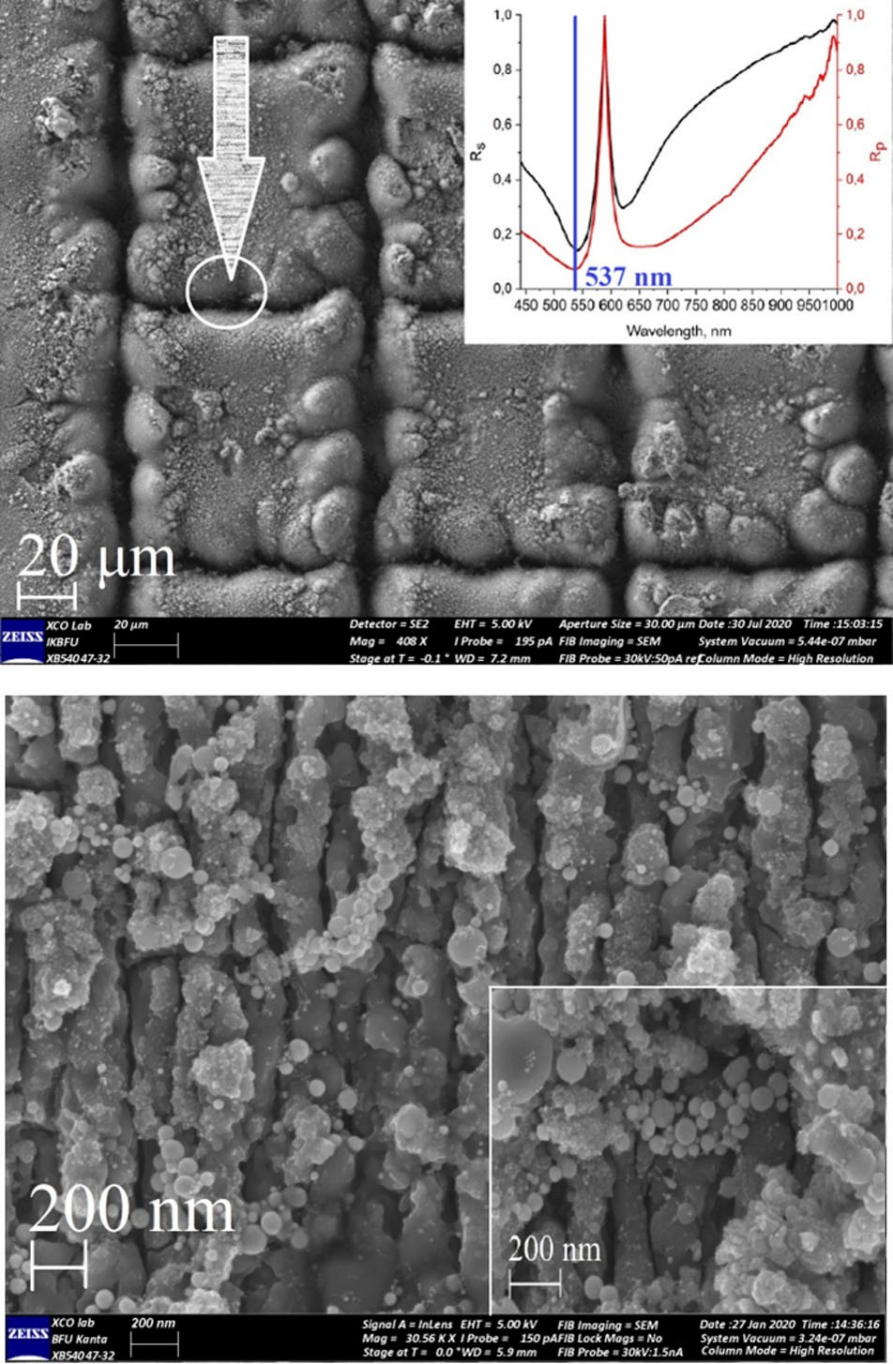
SEM images of the SERS surface in “grating’ geometry on the titanium plate. 20 μm scale (a) with Au NPs and 1 μm scale (b), 200 nm scale (b, inset) with Au NPs. 1a (inset) illustrates results for spectral ellipsometry experiments for Ti/Au surface.

SERS substrate fabrication was performed as three-stage process based on [9, 13]: Firstly, in order to create roughness, anodizing of titanium films with a thickness of 0.1 mm was carried out. Anodizing was performed on the laboratory hand-made equipment with a current source and a galvanic bath, in which titanium electrodes were immersed. An aqueous solution of KOH (5%) was used as the electrolyte. Anodizing was carried out at a current density of *j* = 30 mA for 5 min. The titanium surface became a dark blue color after anodizing process. Further, the anodized titanium surfaces were exposed to femtosecond laser influence. The single mode femtosecond laser (Avesta TETA-25/30 laser system, Russia) (pulse duration of τ = 280 fs and a frequency of ν = 25 kHz) operating at λ = 1032 nm with horizontal linear polarization of laser beam was used for LIPSS formation on anodized Ti plate. As the second step, the anodized titanium plate was mounted on a motorized positioner 8 MTF-102LS05 (Standa, Lithuania), controlled by XILab software. A laboratory-made for XILab software was created to perform the surfaces with “grating”^12^ morphology (Fig 1A). The structures have been fabricated at the following technical conditions: laser spot size was 10 μm; the power of laser radiation in the beam was 200 mW; the average speed scanning during fabrication process was 1000 μm/s. As a last step, gold nanoparticles were deposited on this structured anodized Ti surface (Fig 1B). The gold nanoparticles were prepared also by the femtosecond laser ablation method (Avesta TETA-25/30 laser system, Russia) from the chemically pure (99,99%) gold plate. Each package of laser pulses was focused to a new location of the gold plate. After ablation, the solution assumed slightly red color. The morphology of Ti/Au surfaces was investigated using Carl Zeiss Cross Beam-540 (FIB-SEM) electron microscope. SEM images were taken from the surface both for “gratings” (Fig 1A) and spherical Au NPs, deposited on laser beam path (Fig 1A and 1B (inset)). Spectroscopic ellipsometry experiments were performed (Auto SE (Horiba), France) to investigate plasmon activity of the SERS substrates. The absorption maximum of *p*- and *s*- polarized light was revealed at λ = 537 nm (Fig 1A (inset)). Consequently, we use the excitation source at λ = 532 nm to correlate the excitation with surface *s*- and *p*-polarized light reflection minimums.

**Fig 2:**
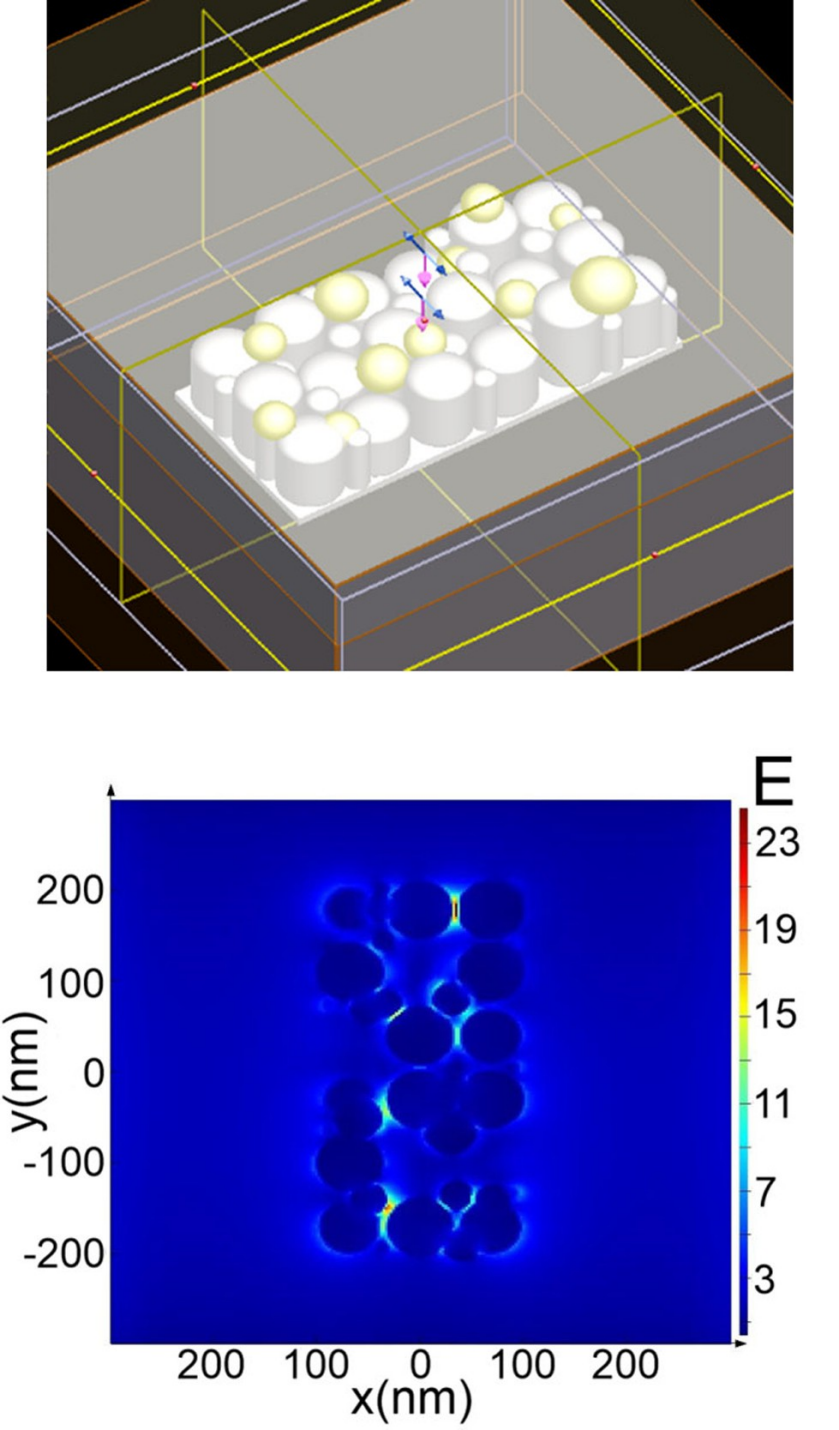
FDTD modeling SERS substrate. FDTD model area with “grating” surface geometry (a) and electric field strength distribution (b).

To evaluate theoretical SERS enhancement factor (EF), (Finite Difference Time Domain) FDTD-based simulation was performed after surfaces fabrication on the “slope” between surfaces of the square and laser path area (Fig 1). Models were created based on the obtained experimental SEM images of the surfaces. The modeling was carried out using the Lumerical FDTD Solutions software package (v.8.19.1584, Lumerical Inc.). Simulation algorithm was based on [13], but we have used Ti as a substrate material, instead Au. Additionally, the 11 model spheres of Au with size D = 15, 20, 25, 30 nm were added into the Ti surface (Fig 2A). Briefly, simulation parameters were set as: the travel time of a plane-polarized wave through the working area as 1000 fs and the temperature of 300°K, Excitation wavelength was set as λ_ex_ = 532 nm. As a final step, we calculated values of the E field in our simulation. It was recalculated into the SERS intensity and |E|^4^ coefficient using FDTD Lumerical scripts. The effective SERS enhancement was defined as |E/E_0_|^4^, where E is the local maximum electric field and E_0_ is the input source for electric field amplitude in linear simulations. We used the Total-Field Scattered-Field (TFSF) source to simulate the electric field on a rough surface and find the maximum possible EF and intensity of the SERS signal.

The maximum overall calculated enhancement factor (EF) was up to 10^3^, depending on the position of the monitor in “hot-spot” zones (Fig 2B and Table 1). Implementation of Au NPs into the simulation area result in E enhancement increasing the overall predicted SERS EF. The greatest perturbation of the electric field arose between cylinders and Au NPs on the Ti surface of the structures in both cases. Taking into account the above-mentioned fact, for better substances surface adsorption and for them to reach hot-spots resulting in high SERS enhancement factors achievement, roughed surfaces should be fabricated.

**Table 1:** Results of SERS surfaces FDTD simulations.

Morphology type/parameters	Local field, *E*, W/m	EF, |E/E_0_|^4^ 10^3^ *xz*	EF, |E/E_0_|^4^ 10^4^ *yz*	SERS signal intensity,a.u. *xz*	SERS signal intensity, a.u. *yz*
“Grating”	24.8	1.2·10^3^	3.65	32	3.0

### SERS experiment

SERS spectra were obtained by Centaur U (LTD «NanoScanTechnology», Russia) Raman spectrometer, using the λ = 532 nm DPSS Cobolt Samba excitation laser with 45 mW power on sample. The optical scheme included Olympus BX 41 microscope with 100X (NA 0.9) objective. Spectrometer Shamrock 750 (Andor, UK) had a focal length of 800 mm and was equipped with 300 gr/mm diffraction grating with 500 nm blaze. IDus 401-DV CCD camera (Andor, UK) with 1024 × 256 pixels sensor was used for the experiments. Spectrometer had spectral resolution of 1.5 cm^-1^. The laser spot of 1 × 25 μm size was positioned at the platelets. Rayleigh scattering was eliminated by the notch filters.

**Fig 3:**
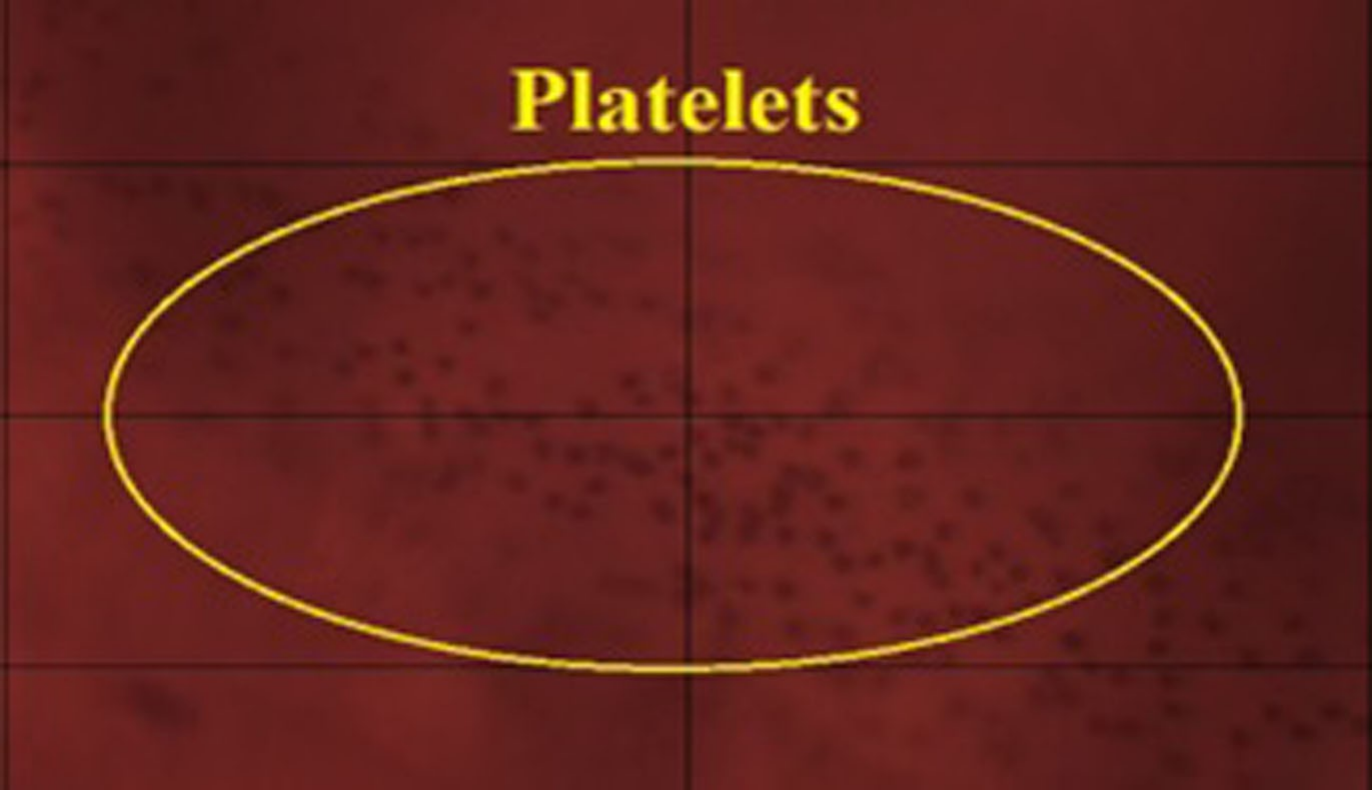
Image of a platelet mass. 100x optical image of the platelet mass, deposited on Ti/Au surface.

5 μl droplet of platelet-rich plasma was put on substrate, dried for 5 minutes at room temperature, and then placed to the microscope holder. Three times averaged spectra from ten different places of the droplet have been collected for each sample. Signal acquisition time was 70 seconds. Each time before experiment, spectrometer was calibrated with silicon at a static spectrum centered at 520.1 cm^−1^ for 1 s. After registration, spectra were saved as .txt and specific format (.ngs) on PC, connected to the Raman unit. Fig 3 illustrates platelet mass, deposited on Ti/Au surface. SERS experimental factor was calculated according Formula 8 in [14] and was up to 1.12⸱10^3^.

### Spectral preprocessing and statistical analysis

Spectra were collected at 200–2000 cm^−1^ spectral range and 400–1800 cm^-1^ range was selected for further spectral and statistical manipulations.

**Fig 4:**
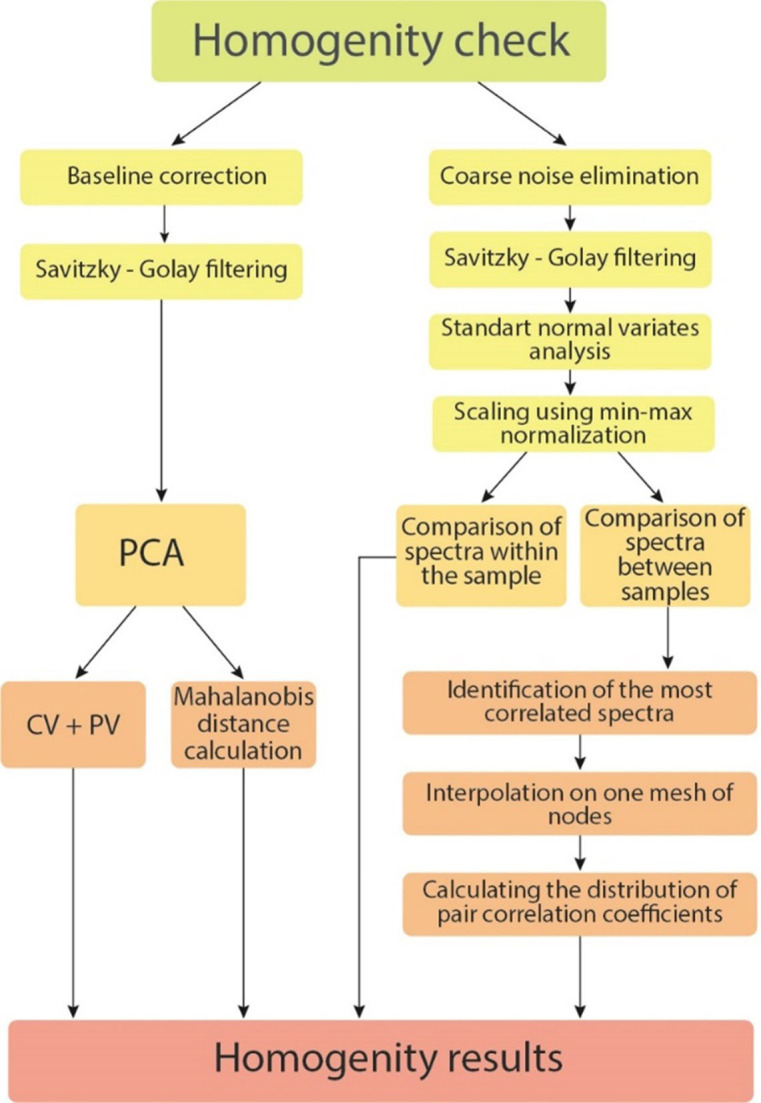
Principal scheme of statistical analysis.

Spectra were collected at 200–2000 cm^−1^ spectral range and 400–1800 cm^-1^ range was selected for further spectral and statistical manipulations. According to Fig 4, we perform two approaches for statistical processing for all 94 spectra. Principal component analysis (PCA) was applied for all spectra classification using KnowItAll Vibrational Spectroscopy Edition (License № 112029) and its MVP statistical module was performed as a first basic approach. Classification methods, based on estimation of their paired correlations values spread, as well as the average moment of these correlations were performed as a second and advanced one. PCA analysis preprocessing included Savitzky-Golay filtering and baseline normalization. The processing procedures based on the second approach included four main steps. Elimination of coarse noise by searching for single knocked out points (single outliers) and replacing them with the average value of six neighboring points were performed first. Suppression of "normal" statistical fluctuations using the Savitzky-Golay filter was applied as a second step. Scaling by min-max normalization within a group of spectra taken from one person was performed as a final preprocessing step. Scaling, which was carried out within the groups of spectra taken from one sample, including the case before and after rebooting the equipment, were performed with two successive procedures: a) Standard Normal Variate (SNV)—for comparability of spectra in intensity. b) Minimum-maximum normalization, since when using SNV the spectra always have positive and negative values centered at 0, and for further analysis it is preferable that the spectra are completely located in the positive/upper half-plane.

## Results and discussion

**Fig 5:**
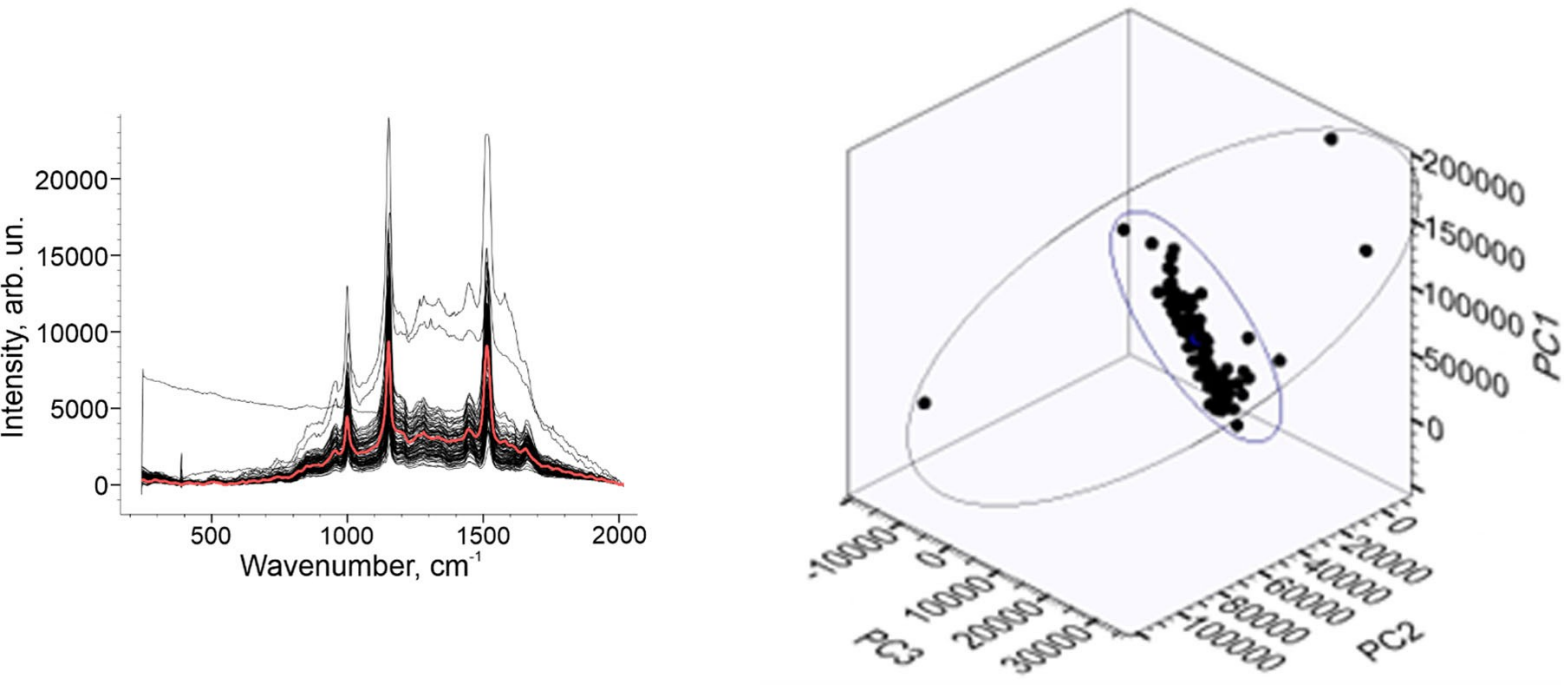
PCA of Raman dataset. Raman spectral dataset (a) and PCA 3D plot of platelets set (b).

The main idea of the paper was to investigate the spectral “similarity”. Spectral correlation can be a fairly simple way to widespread measure for this problem. Performing PCA as a first way, the principal component scores (PC) were set. The output variables (PC1, PC2, PC3) were used to describe spectral differences in the platelet spectral data. Fig 5A illustrates all baseline corrected and filtered Raman dataset. The good spectral uniformity is shown except three anomalous spectra caused by SERS substrate. The averaged Raman spectra marked as red line (Fig 5A). 3D PCA plot with three PC is illustrated on Fig 5B.

**Fig 6:**
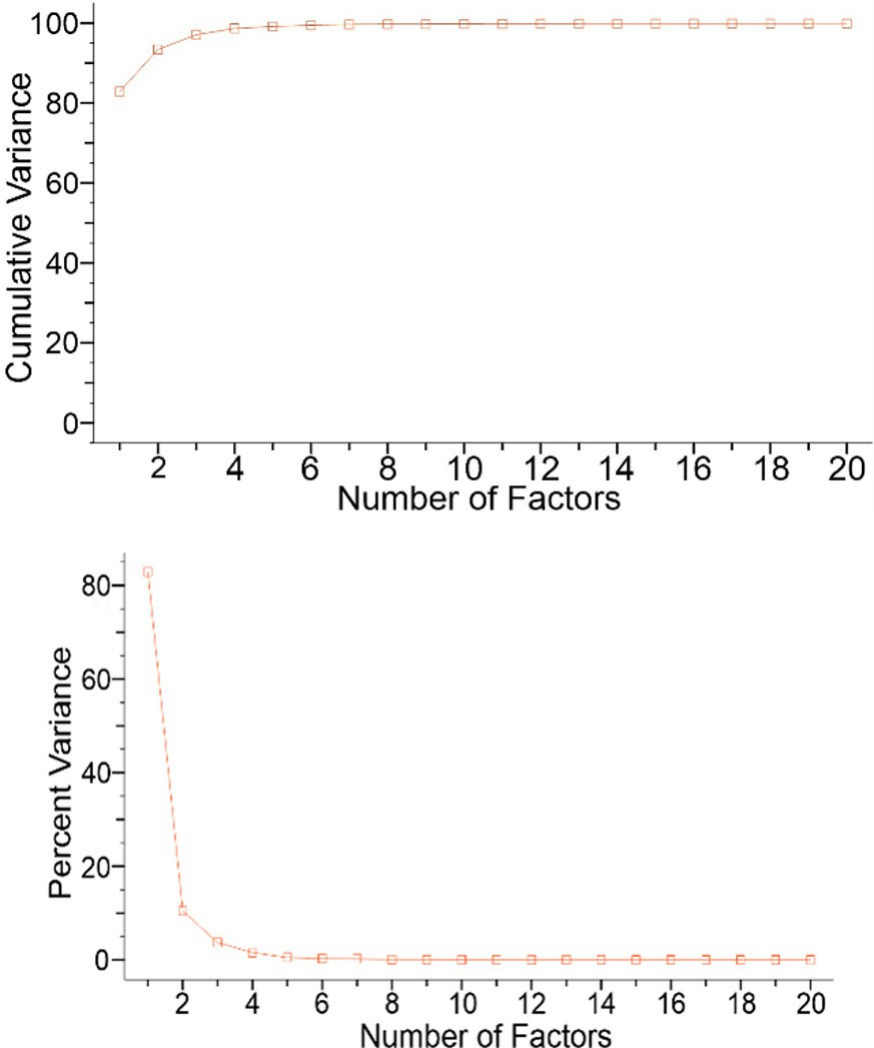
Variances of Raman dataset. Cumulative variance of Raman dataset (a) and Percent variance value (b).

3D plot denotes Raman representation, grouped for the healthy patient. Fig 6 illustrates cumulative variance of Raman dataset (Fig 6A) and Percent variance value (Fig 6B). The first three components are represented by the axes of the plot and capture the most variance of the data. The cumulative variance (CV) of the dataset indicated that principal components PC1 to PC3 are responsible for 97% of all spectral variations: PC1 = 81.8%; PC2 = 93.4%; PC3 = 97.2%. From the other hand, the percent variance (PV) value was 3.5% for the spectral variations: PC1 = 81.27%; PC2 = 12.09% PC3 = 3.29%.

**Fig 7:**
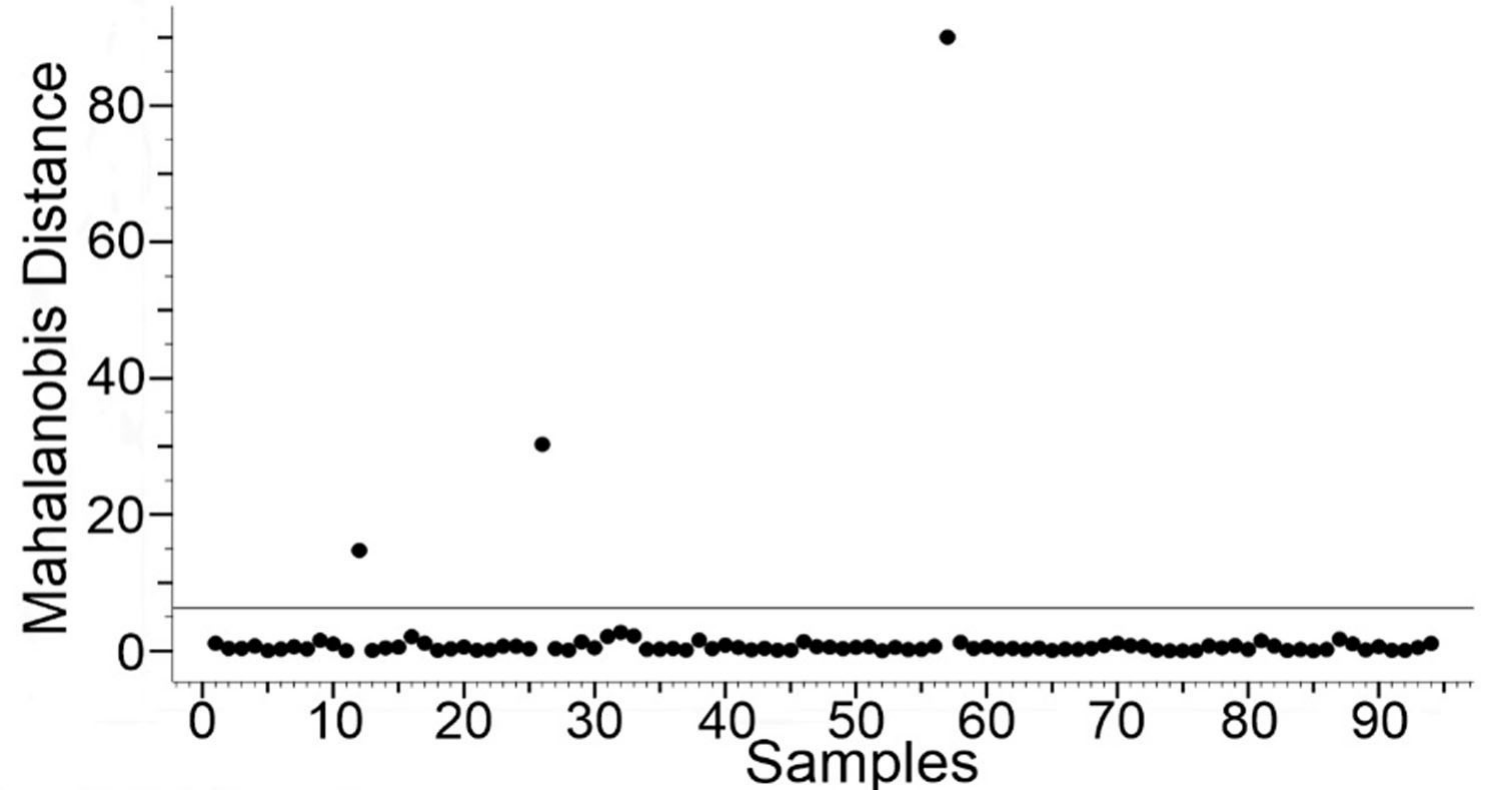
Mahalanobis distance distribution for Raman spectral set.

We used the Mahalanobis distance as a specific parameter inside the spectra to determine the similarity of Raman spectra [15]. Mahalanobis distance takes into account not only the relative distance from the sample but the covariance of the data. It weights the data changes by the range of variability in the direction of the sample point. Mahalanobis distance is measured in terms of standard deviations from the mean of the samples. In that case, the reported matching values give a statistical measure of how well the spectral parameter of an unknown sample matches with the original one [16]. Calculated Mahalanobis distance for 91 spectra was not more than 5 and for three spectra was 14, 32, and 91. It had shown a minimum deviation for 97% of the spectral set. Since Mahalanobis distance approach relies on the reasonable intuition that a good similarity function should assign a large (respectively small) score to pairs of points of the same class, as illustrated in Fig 7 we can conclude that the platelet spectra are highly homogeneous [17].

**Fig 8:**
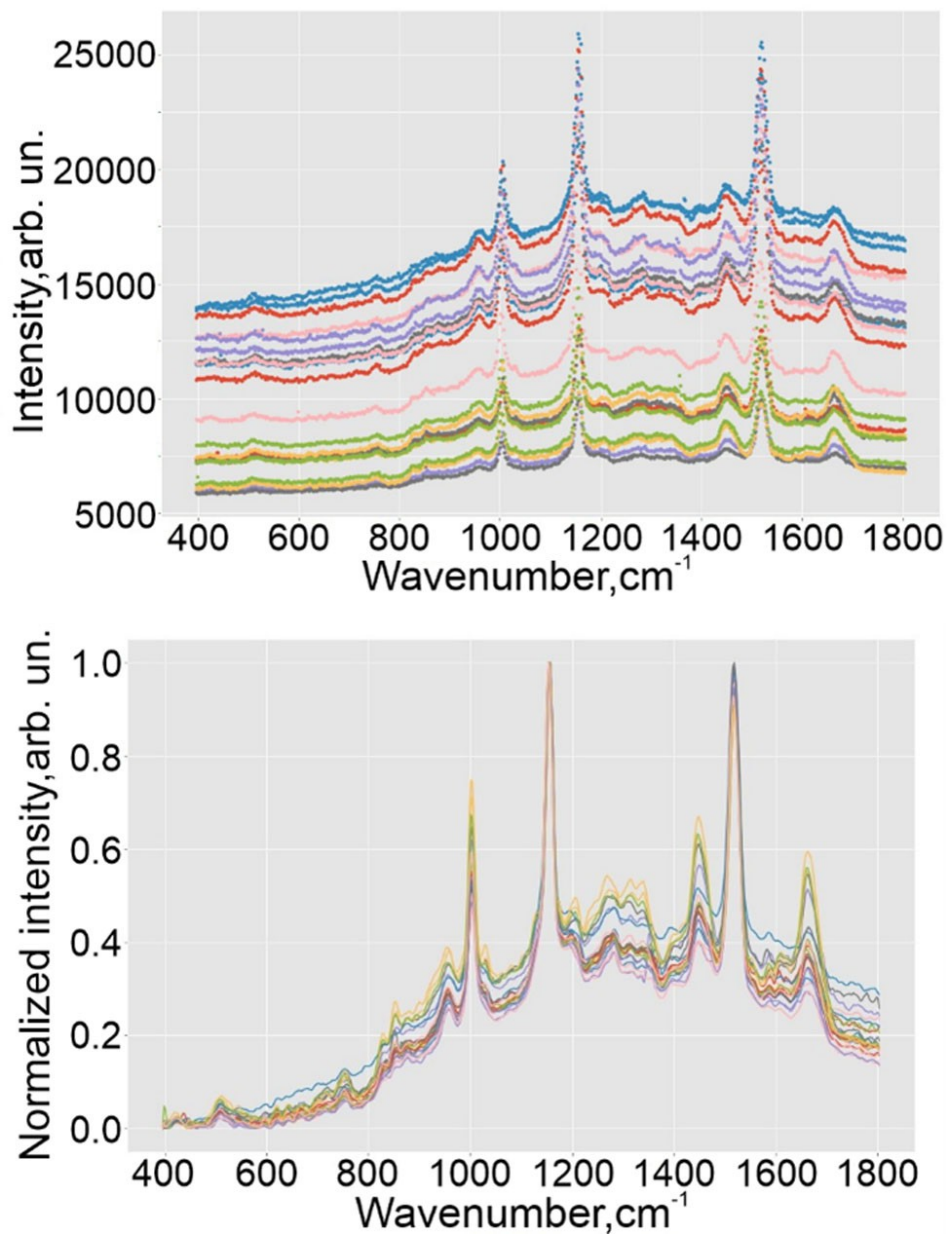
Preprocessing of Raman dataset. Spectral set for the one probe of healthy volunteer before preprocessing (a) and after it (b).

Studying the spectral homogenity deeper, we have implemented the second approach. Two scientific purposes we set: 1) to compare the spectra taken from different points of the one drop. 2) Compare spectra between the different drops taken from one volunteer. Fig 8A illustrates an example of spectral set for the one sample before preprocessing, and Fig 8B after it.

There are different statistical ways to quantify the degree of “similarity” of spectra [18, 19]. Correlation is a fairly simple and widespread measure for this. The coefficients of Pearson, Spearman and its other indicators are used in the form in which they are applied in statistics, as well as their modifications. The formula for the Pearson correlation coefficient is often used for the correction of the mean values of the samples. Using it, it is possible to set their values as zero. In this case, we get the value of the normalized cross-correlation function at zero shifts. Such version of the correlation coefficient is better suited for comparing spectra with an axially aligned baseline. However, as it became clear from our experimental data, that such an indicator gives too high values even with a sufficiently strong visual difference between the spectra, which makes it uninformative. Implementing the second statistical approach, we did not perform the full baseline correction in preprocessing procedures for a number of reasons below (ranked by their importance):

1. Experimental spectra contain a similar background, and there is no need to compare them with spectra without it (for example, with theoretical spectra, as in the problem of substance detection).
2. SNV scaling, correcting the baseline shift and global intensity variations, compares well the experimental spectra, which can be seen, for example, in Fig 8B.
3. Algorithms for adjusting the baseline do not always work correctly.

To assess the similarity, we settled on the Pearson correlation coefficient. Due to the correction for mean values, has the necessary sensitivity to the similarity and difference of the spectra with the background. A value of 0 indicates a complete mismatch of the spectra, and 0.9 and higher in 1, on the contrary, indicates their identity.

**Fig 9:**
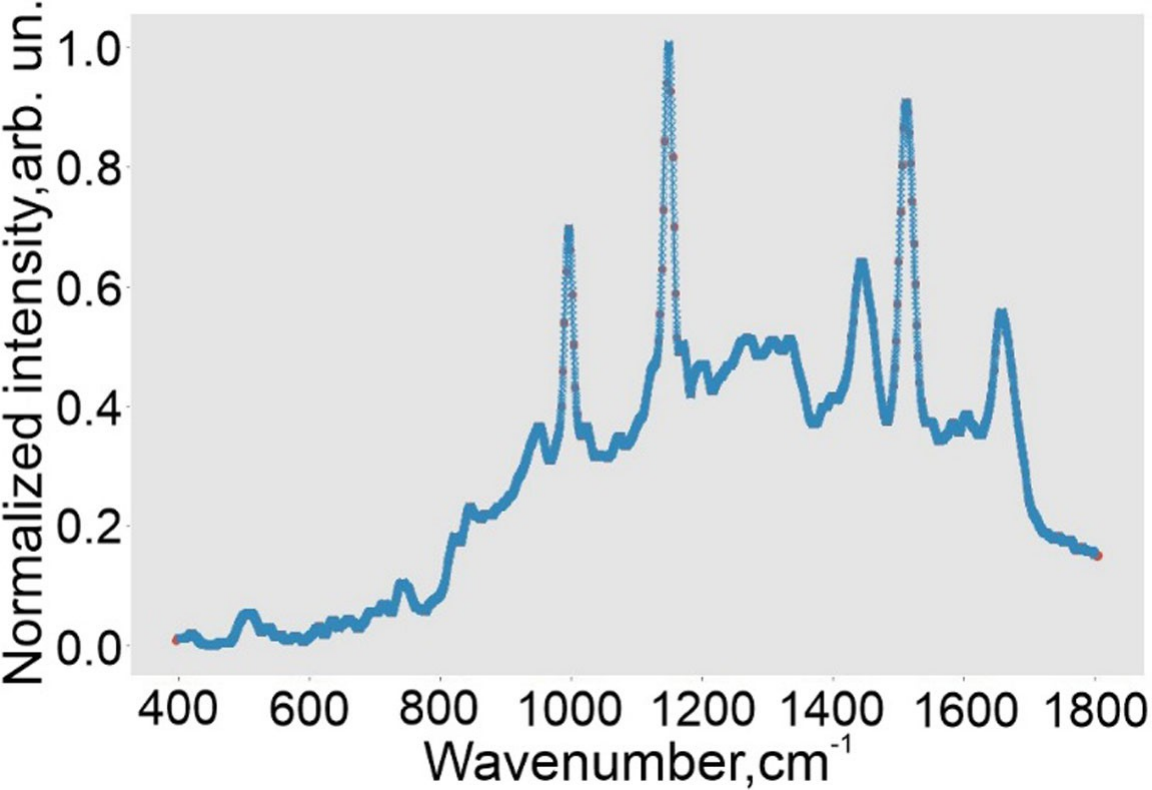
Example of interpolated spectra.

The indicators of the similarity/homogeneity of the spectra of one sample are the scatter of the values of their paired correlations, as well as the average moment of these correlations. In the general case, the correlation coefficient is not additive (since it is not a linear dependence) and its averaging, although arithmetically possible, does not coincide with the average correlation among all samples. Instead, usage of another, additive, indicator of dependence, usually it is a coefficient of determination equal to the square of the correlation coefficient. However, given the nature of the data under consideration, as well as the fact that none of the correlation coefficients calculated for this study takes a negative value, their averaging will be an adequate average measure. The hypothesis of spectral homogeneity of platelets obtained on different days from one healthy person was tested in three stages. First, among the spectra of one sample, the most correlated with the others was revealed, i.e., the sum of its correlation coefficients with them exceeded similar ones. It means that there was a row in the correlation matrix, the sum of which was the largest. (The same sense would be to find the average value instead of the sum). The best spectra found in this way from each of the samples were compared, just as it was with the spectra taken from one sample. But in contrast to them, in the spectra of different samples, the coordinates of the points do not coincide along the frequency axis—they have a different step and shift. Therefore, in order to compare the best spectra from each sample in one grid of nodes, for each of them, using cubic interpolation, the intensity values were calculated at 5000 (new) points, the value of which along the frequency axis evenly covers the spectral range of 400–1800 cm^-1^. An example of the initial points of one spectrum and obtained using the interpolation function is shown in Fig 9.

**Fig 10:**
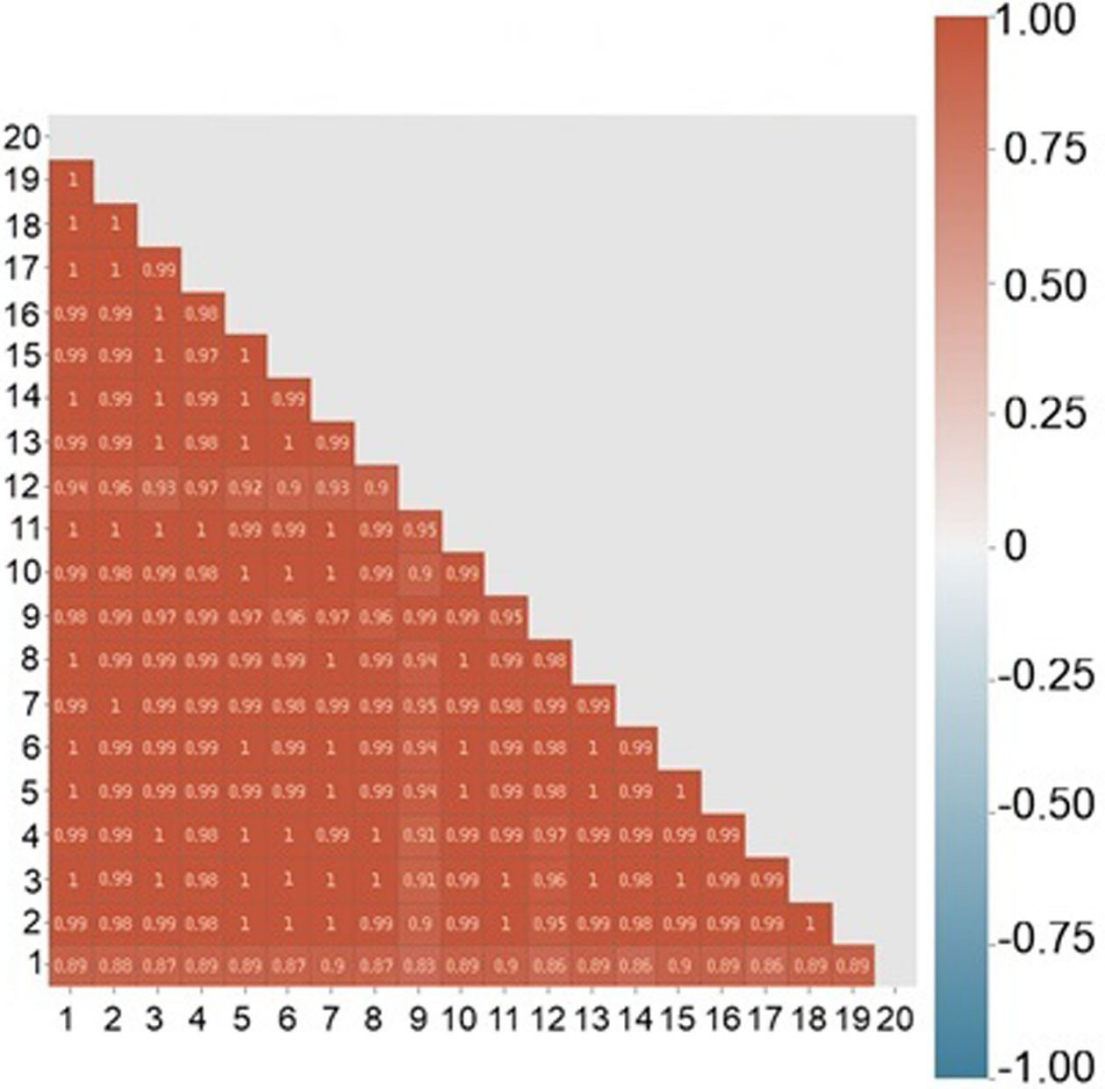
Raman spectra pair linear spectral correlation inside one sample.

**Fig 11:**
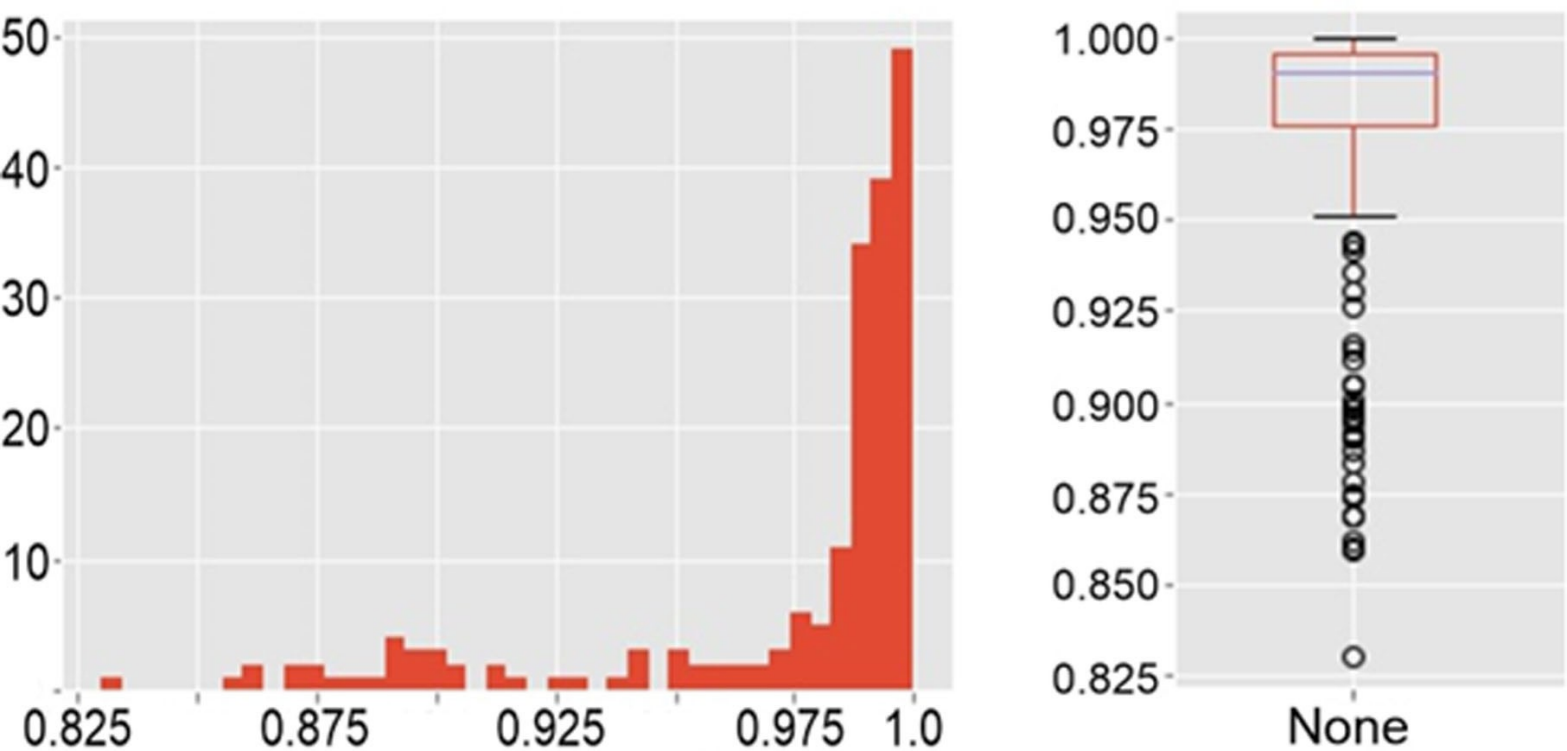
The distribution of values. A histogram (a) and a boxplot (b).

For each sample, the correlation of all pairs of its spectra was considered. In Fig 10 shows a matrix of correlations compiled for 20 spectra at different points of one sample of a healthy person, half of which were taken after restarting the equipment. Fig 11 shows the distribution of values from this matrix is shown in Fig 10 as histogram (Fig 11A) and boxplot (Fig 11B).

**Fig 12:**
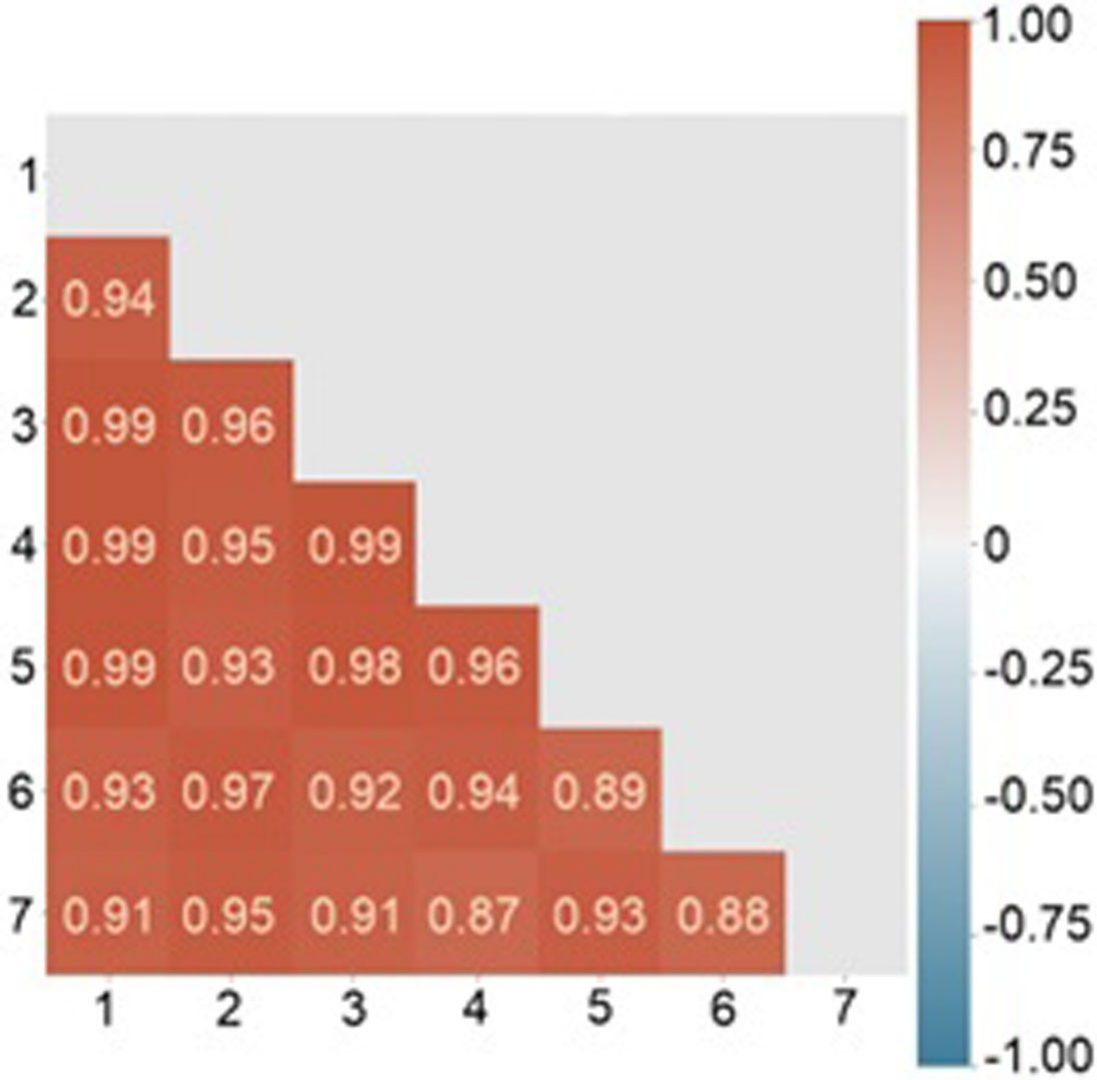
Raman spectra pair linear spectral correlation between samples.

**Fig 13:**
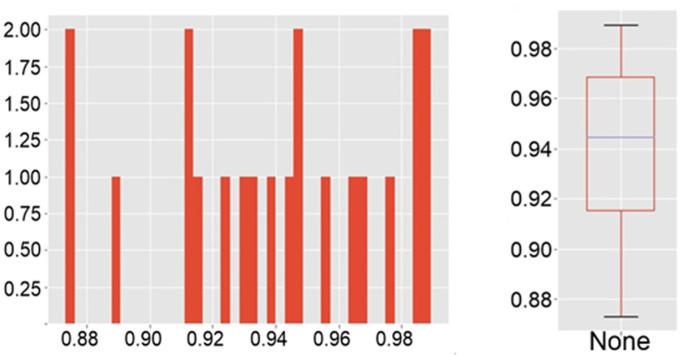
The distribution of values. A histogram (a) and a boxplot (b).

To analyze the correlation between samples all pairs of their spectra for each sample were considered and compared. Fig 12 shows a matrix of correlations compiled for 7 sets consisted of 10 spectra each. All the spectra were taken in different points of one sample of a healthy person. The distribution of values is shown as a matrix (Fig 12), histogram (Fig 13A) and boxplot (Fig 13B).

The arithmetic mean of the correlation coefficients for each sample was more than 0.92, and the average coefficient of determination was more than 0.86. The average measures have high values for the sample, the spectra of which were taken with the reboot of the equipment. Moreover, despite the fact that the minimum value of the correlation between the two spectra (0.60122) is in this particular sample, already the 15th percentile for it, as well as for all other samples, has a value was more than 0.9. Thus, it can be argued that the spectra of one sample are homogeneous, including they are reproduced even after the equipment is rebooted; only a small number (up to 15%) have significant deviations—possibly due to the fact that residual erythrocytes, damaged/degrading platelets, and other reasons could appear at the shooting point. Our results can be compared with heparin-induced thrombocytopenia (HIT) research using SERS [20]. Authors use SERS and statistical approaches to discriminate HIT positive and HIT negative samples. Our approach correlates with [21] and we can state that platelet spectral homogeneity can be used as tool for differentiation of patients in different conditions.

## Conclusions

In this paper we have revealed the spectral homogeneity of human platelets taken from healthy volunteer. We have performed combined approach based on multivariate methods as principal component analysis and pair correlation algorithms to investigate platelet’s spectral properties. The high degree of spectral homogeneity inside one probe and between them has been revealed. The significant prospects of using platelet spectra for pathologies study based on platelet conformations during cardiovascular diseases have been demonstrated.
